# Effect of temporary cements on the shear bond strength of luting cements

**DOI:** 10.1590/S1678-77572010000100007

**Published:** 2010

**Authors:** Marco FIORI-JÚNIOR, Wilson MATSUMOTO, Raquel Assed Bezerra SILVA, Sizenando Toledo PORTO-NETO, Jaciara Miranda Gomes SILVA

**Affiliations:** 1 DDS, MSc, Ribeirão Preto Dental School, University of São Paulo, Ribeirão Preto, SP, Brazil.; 2 DDS, MSc, PhD, Professor, Department of Dental Materials and Prosthodontics, Ribeirão Preto Dental School, University of São Paulo, Ribeirão Preto, SP, Brazil.; 3 DDS, MSc, Professor, Department of Pediatric Clinics, Preventive and Community Dentistry, Ribeirão Preto Dental School, University of São Paulo, Ribeirão Preto, SP, Brazil.; 4 DDS, MSc, PhD, Professor, Department of Operative Dentistry, Araraquara Dental School, São Paulo State University, Araraquara, SP, Brazil.; 5 DDS, Doctoral student, Department of Pediatric Clinics, Preventive and Community Dentistry, Ribeirão Preto Dental School, University of São Paulo, Ribeirão Preto, SP, Brazil.

**Keywords:** Shear bond strength, Temporary cementation, Resin-based luting cements

## Abstract

**Objective:**

The purpose of this study was to evaluate, by shear bond strength (SBS) testing, the influence of different types of temporary cements on the final cementation using conventional and self-etching resin-based luting cements.

**Material and Methods:**

Forty human teeth divided in two halves were assigned to 8 groups (n=10): I and V (no temporary cementation); II and VI: Ca(OH)_2_-based cement; III and VII: zinc oxide (ZO)based cement; IV and VIII: ZO-eugenol (ZOE)-based cement. Final cementation was done with RelyX ARC cement (groups I to IV) and RelyX Unicem cement (groups V to VIII). Data were analyzed statistically by ANOVA and Tukey's test at 5% significance level.

**Results:**

Means were (MPa): I - 3.80 (±1.481); II - 5.24 (±2.297); III - 6.98 (±1.885); IV - 6.54 (±1.459); V - 5.22 (±2.465); VI - 4.48 (±1.705); VII - 6.29 (±2.280); VIII - 2.47 (±2.076). Comparison of the groups that had the same temporary cementation (Groups II and VI; III and VII; IV and VIII) showed statistically significant difference (p<0.001) only between Groups IV and VIII, in which ZOE-based cements were used. The use of either Ca(OH)_2_ based (Groups II and VI) or ZO-based (Groups III and VII) cements showed no statistically significant difference (p>0.05) for the different luting cements (RelyX^TM^ ARC and RelyX^TM^ Unicem). The groups that had no temporary cementation (Groups I and V) did not differ significantly from each other either (p>0.05).

**Conclusion:**

When temporary cementation was done with ZO- or ZOE-based cements and final cementation was done with RelyX ARC, there was an increase in the SBS compared to the control. In the groups cemented with RelyX Unicem, however, the use of a ZOE-based temporary cement affected negatively the SBS of the luting agent used for final cementation.

## INTRODUCTION

With the development of enamel-dentin etching techniques and resin luting agents for cementation of prosthetic pieces, adhesive cementation techniques have been used not only for metal-free dentures, but also for partial orer complete metal crowns^2^. Theoretically, luting cements present some advantages when used for final cementation, due to physical characteristics, such as insolubility in oral fluids, high bond strength to dentin and enamel, thin cement film and good esthetics^9,18^. These materials are classified according to their setting reaction as chemically activated, light-activated and dual activated luting cements^7^. Several clinical studies have reported the long-term success of indirect restorations bonded with resin cements, including ceramic laminates^8,12^, inlays/ onlays^24^, partial fixed prostheses^10,29^ and complete ceramic crowns^19,20^.

The clinical success of all-ceramic restorations is influenced by the type of luting agent and technique for definitive cementation. The main influencing parameter seems to be adequate adhesion between ceramic restoration and the supporting tooth structures^5^. When a durable and high-quality bonding is obtained between the dental substrate and the prosthetic crown, there is better retention and marginal adaptation, which prevents microleakage and increases fracture resistance of the restored teeth and indirect restorations^25^.

Bonding technology of all-ceramic restorations is generally complicated and furthermore, most all-ceramic techniques require dental laboratory work. This means that a temporary restoration is necessary in order to avoid sensitivity, infection and tooth movement. Zinc oxide-eugenol (ZOE) temporary luting cements are commonly used because of their sedative effect on sensitive teeth. Like other phenolic compounds, eugenol is a radical scavenger, which inhibits the polymerization of resin materials^1^. Contradictory findings have been published with regard to the bond strength to dentin after placement of temporary cements^3,13,23^. In recent years, the increasing demand for all-ceramic restorations led to development of ceramic materials, which require resin bonding for clinical success. Therefore, the purpose of this study was to evaluate, by SBS testing, the influence of different types of temporary cements on the final cementation using conventional (RelyX ARC) and self-etching (RelyX Unicem) luting cements.

## MATERIAL AND METHODS

This study was approved by the Research Ethics Committee of the Ribeirão Preto Dental School, University of São Paulo, Brazil.

Forty healthy freshly extracted human third molars (from the Human Tooth Bank of the Ribeirão Preto Dental School, University of São Paulo, Brazil) stored in distilled water 4°C were used. The teeth had their roots removed 3 mm below the cementoenamel junction with a watercooled diamond saw (Minitom, Struers A/S, Copenhagen, Denmark). The crowns were fixed with wax in Plexglass^®^ plates and bisected longitudinally in a buccolingual direction using a double-faced diamond disk (KG Sorensen, 7015, Barueri, SP, Brazil) mounted in a low-speed handpiece, thus providing 80 halves. The halves were embedded in chemically activated polyester resin into polyvinyl chloride (PVC™) rings (2.1cm diameter and 1.1-cm height), in such a way that their mesial-distal surfaces were faced up. After resin polymerization, the rings were discarded and the surfaces of the teeth were ground with water-cooled #180- to #400-grit silicon carbide (SiC) papers (Buehler Ltd., Lake Bluff, IL, USA) on a polishing machine (Politriz DP-9U2; Struers, A/S) to remove the overlying enamel and expose flat dentin surface. To warrant the complete removal of enamel, the ground surfaces were viewed with a magnifying glass at ×20. Additional wet grinding with #600-grit SiC paper was done for 30 s to produce a standard smear layer. A bonding site was demarcated by attaching a piece of insulating tape with a 3-mmdiameter central hole to each dentin surface. Bonding site delimitation had a double aim: to define a fixed test surface area and to warrant that the resin composite cones could be further adhered precisely to treated dentin surface, thus avoiding accidental adhesion to the surrounding enamel.

The specimens were randomly assigned to 8 groups (n=10), according to the temporary cements used: Groups I and V (controls): no temporary cementation; Groups II and VI: temporary cementation with calcium hydroxidebased temporary cement (Hydro C^®^; Dentsply Indústria e Comércio Ltda., Petrópolis, RJ, Brazil); Groups III and VII: temporary cementation with ZO-based temporary cement (RelyX^TM^ Temp NE; 3M/ESPE, St. Paul, MN, USA); Groups IV and VIII: temporary cementation with ZOE-based temporary cement (Temp Bond^®^; Kerr Corporation, Orange, CA, USA).

For temporary cementation (Groups II to IV and VI to VIII), 60 acrylic resin discs (3 mm in diameter x 2 mm high) (Dencor Acrílico Autopolimerizante; Clássico Artigos Odontológicos Ltda.; São Paulo, SP, Brazil) were fabricated using polytetrafluoroethylene molds with same dimensions.

The temporary cements (Hydro C^®^, Temp Bond^®^ and RelyX^TM^ Temp NE) were prepared according to the manufacturers' instructions. The acrylic discs were positioned on the cement layer and the discs were subjected to 1 kgf (10N) constant load for 2 minutes applied by a universal testing machine (DL 2000; EMIC 2003; São José dos Pinhais, PR, Brazil) during cement setting. Next, the specimens were stored in distilled water at 37°C for 24 h. The acrylic discs were detached to the bonding site by means of a knife-edge blade in the universal testing machine (Mod. MEM 2000; EMIC Ltda) at a crosshead speed of 0.5 mm/min with a 50 kgf load cell. The remaining temporary cement was removed from dentin surface using a hand excavator. The excavator was used with very close (mostly overlapping) parallel strokes under moderate pressure and the procedure was repeated in an overlapping direction if any trace of cement was detected macroscopically. All procedures were performed by a single researcher.

Then, feldspathic ceramic discs with the same dimensions as those of the acrylic discs (3 mm diameter x 2 mm high) were cemented with RelyX^TM^ ARC (3M/ESPE) in Groups I to IV and RelyX^TM^ Unicem (3M/ESPE) in Groups V to VIII, according to the manufacturers' instructions. The cements were carefully applied with disposable microbrush tips (Microbrush Corporation, Grafton, WI, USA) to avoid excess and pooling of adhesive along the edges of the insulating tape that could compromise the distribution of tension during the SBS test and hence the validity of results. For the RelyX^TM^ ARC groups the dentin was etched with a 35% phosphoric acid gel (3M/ ESPE) for 15 s, rinsed thoroughly for 15 s and excess water was blotted with absorbent paper. With a fully saturated brush tip, 2 consecutive coats of an adhesive system (Adper Single Bond; 3M/ESPE) were applied to the tooth and polymerized with a halogen light-curing unit (XL 3000; 3M/ESPE) for 20 s with intensity of 800 mW/cm². A dual-cured resin-based cement (Rely X ARC; 3M/ESPE) was then dispensed onto a mixing pad and mixed for 10 s. A thin layer of the material was applied to the dentin surface, which was seated in place. Cement excess was removed with a microbrush and was polymerized from each face for 40 s. For the RelyX^TM^ Unicem groups the cement was mixed for 10 s, a thin layer of the material was applied to the dentin surface. Resinous cement excess was removed with a brush and was polymerized from each face for 40 s, according to manufacturers' recommendations without any primer or adhesive. After cementation, all specimens were stored in distilled water at 37°C for 24 h. Thereafter, SBS testing was done using a knifeedge blade in the universal testing machine (Mod. MEM 2000; EMIC Ltda) running at a crosshead speed of 0.5 mm/min with a 50 kgf load cell. SBS mean values were recorded in kgf/cm and converted into MPa. Data were analyzed statistically by one-way ANOVA and Tukey's posthoc test using GraphPad Prism^®^ statistical software (version 3.02; Graphpad Software, San Diego, CA, USA) at 5% significance level.

## RESULTS

The SBS mean values in MPa (2.47 to 6.98 MPa range) and standard deviation for all groups are given on Table 1.

**Table 1:** Shear strength mean values in MPa (±SD) for Groups I to VIII

**Specimen**	**Group I**	**Group II**	**Group III**	**Group IV**	**Group V**	**Group VI**	**Group VII**	**Group VIII**
**1**	2.77	1.68	6.97	5.16	2.29	5.06	2.75	3.77
**2**	3.25	7.62	8.34	5.54	2.42	7.40	3.88	5.65
**3**	7.23	1.15	4.10	4.46	7.29	5.26	6.51	5.68
**4**	4.35	6.10	5.18	6.77	2.47	3.61	9.83	3.17
**5**	4.78	5.50	8.45	6.08	5.22	3.30	5.43	1.77
**6**	3.79	5.24	9.79	7.40	4.83	1.44	6.20	0.29
**7**	2.75	6.15	6.99	8.58	9.24	6.45	9.25	0.17
**8**	4.15	6.31	6.97	5.83	4.02	4.48	4.60	2.47
**9**	2.18	4.38	5.01	6.54	6.56	3.29	8.16	1.30
**10**	2.57	8.22	8.03	9.04	7.84	4.48	6.30	0.40
**Mean**	3.80	5.24	6.98	6.54	5.22	4.48	6.29	2.47
	(±1.481)	(±2.297)	(±1.885)	(±1.459)	(±2.465)	(±1.705)	(±2.280)	(±2.076)

**Figure 1:**
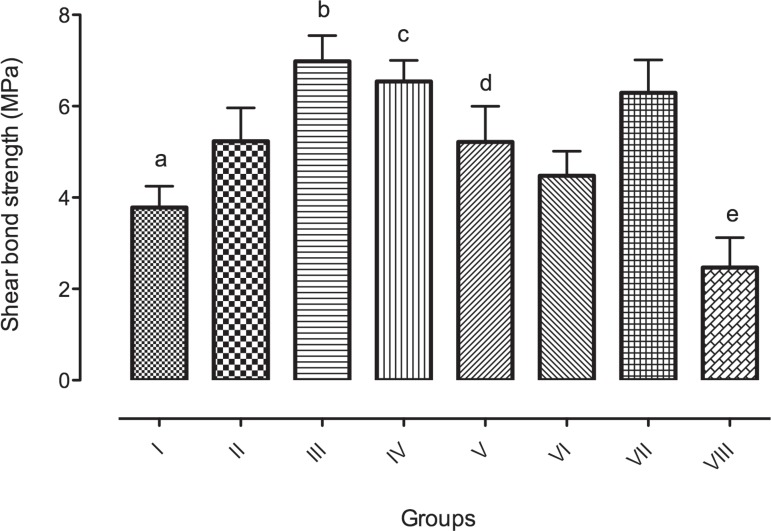
Shear bond strength mean values (in MPa) for Groups I to VIII. Different letters indicate statistically significant difference (α=0.05, please refer to text)

Regarding Groups I to IV, there was no statistically significant difference (p>0.05) between the Groups I (no temporary cementation and final cementation with RelyX^TM^ ARC) and II (temporary cementation with Ca(OH)_2_-based cement and final cementation with RelyX^TM^ ARC). There was, however, significant difference (p<0.01) between Group I and the other groups in which temporary cementation was performed (Groups III and IV). Group II did not differed significantly (p>0.05) from Groups III (temporary cementation with ZO-based cement and final cementation with RelyX^TM^ ARC) and IV (temporary cementation with ZOE-based cement and final cementation with RelyX^TM^ ARC). Likewise, there was no significant difference (p>0.05) between Groups III and IV (Figure 1).

Regarding Groups V to VIII, statistically significant difference (p<0.05) was found between Groups V (no temporary cementation and final cementation with RelyX^TM^ Unicem) and VIII (temporary cementation with ZOE-based cement and final cementation with RelyX^TM^ Unicem). Group V did not differ significantly (p>0.05) from Groups VI (temporary cementation with Ca(OH)_2_-based cement and final cementation with RelyX^TM^ Unicem) and VII (temporary cementation with ZO-based cement and final cementation with RelyX^TM^ Unicem). In the same way, there was no significant difference (p>0.05) when Group VI was compared to Groups VII and VIII. However, Groups VII and VIII differed significantly from each other (p<0.01) (Figure 1).

Comparison of the groups that had the same temporary cementation (Groups II and VI; III and VII; IV and VIII) showed statistically significant difference (p<0.001) only between Groups IV and VIII, in which ZOE-based cements were used. The use of either Ca(OH)_2_-based (Groups II and VI) or ZO-based (Groups III and VII) cements showed no statistically significant difference (p>0.05) for the different luting cements (RelyX^TM^ ARC and RelyX^TM^ Unicem). The groups that had no temporary cementation (Groups I and V) did not differ significantly to each other as well (p>0.05).

## DISCUSSION

Luting agents comprise a wide array of materials used for fixation of crowns and indirect restorations to prepared teeth^2^. Because of the better mechanical properties and greater retention ability of resin-based agents^9,18^, their use has increased considerably in the last years.

To simplify tooth-conditioning procedures, recently, the concept of self-adhesive cement has been launched into the market. Self-adhesive resin cements are claimed to provide good bond strengths to tooth structures and restorative materials without any pretreatment or bonding agents. Therefore, their application is very simple and can be accomplished in a single clinical step, similar to conventional luting agents, such as zinc phosphate and glass ionomer cements^5^. In the present research, Rely X Unicem self-adhesive cement, a dual-cure powder and liquid material, was used. Highest SBSs were obtained for Groups I and V, in which RelyX^TM^ ARC (3.80 MPa) and RelyX^TM^ Unicem (5.22 MPa) were used, respectively, without temporary cementation. Ernst et al.^11^ assessed *in vitro* the bond strength of 4 luting cements, including the RelyX^TM^ Unicem. The median (minimum/maximum) bond strength values for this cement were 4.8 (2.5/6.7) MPa. In the present study, the group cemented with RelyX^TM^ Unicem (Group V) had mean shear strength of 5.22 MPa.

The results of Groups II and VI (temporary cementation with a calcium hydroxide-based cement) indicated that this material did not affect the bond strength to dentin of both luting cements used for final cementation, which is consistent with the findings of a previous work^13^. However, these results disagree with those of Paul and Scharer^21^, who reported that the use of Ca(OH)_2 _based cements for temporary cementation reduced the bond strength to dentin of the luting agents used for final cementation.

In Groups III and VII (temporary cementation with eugenol-free zinc oxide cement), bond strength means (6.98 and 6.29 MPa) were lower than those reported in previous studies^26,28^. However, the results of the present study showed that the use of ZO-based temporary cements did not affect adversely the bond strength to dentin of either the conventional (RelyX^TM^ ARC) or the self-etching (RelyX^TM^ Unicem) luting agents used for final cementation, which is consistent with the findings of other studies^1,21^.

There are controversial results referring to the use of ZOE-containing temporary cements. Some authors advocate that these materials present a good clinical performance; in addition to have different biologic properties, depending on their concentration, the presence of eugenol provides a "sedative" effect on the pulp^1,6,21,31^. Eugenol is able to penetrate and diffuse throughout the dentin^6,17^. After release, its diffusion rate increases and reaches its peak within 24 h of contact with dentin, decreasing slowly after 14 days^6^. It is also known that the polymerization of resin-based materials and adhesive systems is induced by chemical- or light-activated radicals. The hydroxyl group of eugenol tends to protonate these radicals and block this reactivity^14^. This fact has led to the development of several studies^1,4,13,16,21,22,23,31^ to assess the influence of eugenol-containing temporary cements on the bond strength to dentin of adhesive systems and luting cements. Some of these studies confirmed that eugenol inhibited the polymerization of resin materials^3,4,14,21,27,31^ while other studies reported that the use of eugenol-containing temporary cements had no adverse effect on the polymerization of the tested materials^1,13,22,23,26^.

Woody and Davis^30^ suggested that the detrimental effect on bond strength to dentin in specimens that have been primarily subjected to temporary cementation may not be caused by the eugenol, but rather by the presence of temporary cement remnants. These remnants have been observed microscopically on macroscopically clean surfaces^27,28^. Different methods for removal of temporary cement remnants have been investigated, including air abrasion^1^, water/pumice prophylaxis^21,31^, ultrasound^30^ and mechanical removal with curettes^1,13^. In the present study, the temporary cement remnants were mechanically removed with excavators, based on the findings of a previous study^1^, which showed that there are no significant adverse effects on the bond strength of ceramics to dentin when either air abrasion or curettes were used for cleaning.

The results of the present study showed that the influence of eugenol-containing temporary cements may depend on the composition of the luting agent used for final cementation. In Group IV (Temp Bond^®^ eugenol-containing temporary cement plus RelyX^TM^ ARC), bond strength to dentin was not adversely affected, which is in agreement with the findings of several studies^1,13,22,23,26^. On the other hand, Group VIII (Temp Bond^®^ eugenol-containing temporary cement plus RelyX^TM^ ARC) had statistically significant lower bond strength. This suggest that the eugenol-containing temporary cement affected adversely the bond strength of the luting cements to dentin, as reported by other studies^3,14,21,27,31^ reported that the eugenol may interfere with the resin polymerization, depending on eugenol concentration in the zinc oxide mixture. The use of non-eugenol or eugenolcontaining temporary cements is a controversial subject.

The results of the present showed that that the presence of eugenol may either increase or reduce the bond strength of final cementation, depending on the composition of the resin luting cement used. Therefore, from a clinical standpoint, it is to investigate the consequences of this interaction for the different types and commercial brands of luting cements available in the market. Further research is required with other materials indicated for temporary and final cementation, including *in vivo* studies and clinical trials. As far as luting agents for cementation of indirect restorations are concerned, general dentists and prosthesists have several options^15^. However, none of the currently available luting agents fulfill all requirements to be considered as the ideal material for any clinical situation. Therefore, the choice for luting cement should be sensible and based on scientific evidence.

The present *in vitro* study assessed the effect of temporary cements on the SBS of final cementation with conventional (RelyX ARC) and self-etching (RelyX Unicem) luting cements to permanent teeth dentin. Nevertheless, it is important to highlight that the lack of studies testing the same methodology and materials in this substrate was a hindrance to stating a reliable comparison between the outcomes of the conducted research and the available data.

## CONCLUSION

Based on the findings of this study and within the limitations of an *in vitro* investigation, the following conclusions can be drawn: 1. when temporary cementation was done with ZO- or ZOE-based cements and final cementation was done with RelyX ARC, there was an increase in SBS compared to control; 2. in the groups cemented with RelyX Unicem, the use of a ZOEbased temporary cement affected negatively the SBS of the luting agent used for final cementation.
